# The effect of relative air humidity on the evaporation timescales of a human sneeze

**DOI:** 10.1063/5.0102078

**Published:** 2022-07-07

**Authors:** Bernhard Stiehl, Rajendra Shrestha, Steven Schroeder, Juanpablo Delgado, Alexander Bazzi, Jonathan Reyes, Michael Kinzel, Kareem Ahmed

**Affiliations:** 1 University of Central Florida, 4000 University Blvd., Orlando, Florida 32816, USA; 2 Propulsion and Energy Research Laboratory, 12781 Ara Dr., Orlando, Florida 32816, USA; 3 Computational Fluids and Aerodynamics Laboratory, 12760 Pegasus Blvd., Orlando, Florida 32816, USA

## Abstract

The present paper investigates droplet and aerosol emission from the human respiratory function by numerical and experimental methods, which is analyzed at the worst-case scenario, a violent sneeze without a face covering. The research findings develop the understanding of airborne disease transmission relevant to COVID-19, its recent variants, and other airborne pathogens. A human sneeze is studied using a multiphase Computational Fluid Dynamics (CFD) model using detached eddy simulation coupled to the emission of droplets that break up, evaporate, and disperse. The model provides one of the first experimental benchmarks of CFD predictions of a human sneeze event. The experiments optically capture aerosols and droplets and are processed to provide spatiotemporal data to validate the CFD model. Under the context of large random uncertainty, the studies indicate the reasonable correlation of CFD prediction with experimental measurements using velocity profiles and exposure levels, indicating that the model captures the salient details relevant to pathogen dispersion. Second, the CFD model was extended to study the effect of relative humidity with respect to the Wells curve, providing additional insight into the complexities of evaporation and sedimentation characteristics in the context of turbulent and elevated humidity conditions associated with the sneeze. The CFD results indicated correlation with the Wells curve with additional insight into features, leading to non-conservative aspects associated with increased suspension time. These factors are found to be associated with the combination of evaporation and fluid-structure-induced suspension. This effect is studied for various ambient air humidity levels and peaks for lower humidity levels, indicating that the Wells curve may need a buffer in dry climates. Specifically, we find that the increased risk in dry climates may be up to 50% higher than would be predicted using the underlying assumptions in Wells’ model.

## I. INTRODUCTION TO AIRBORNE TRANSMISSION STUDIES

The ongoing COVID-19 pandemic has driven a need for research focus associated with the dispersion of respiratory droplets in daily life situations. This continues to be an issue due to the increasing number of vaccine breakthrough cases along with the occurrence of emerging virus variants of COVID-19. It is generally regarded that airborne transmission paths are associated with droplets and aerosols formed from sneezes, coughs, speech, and breathing.^1,3^ A sneeze is the more violent event and creates more droplets relative to other respiratory activities,^2,4^ such as speaking or coughing, and is often regarded as the worst-case scenario of the human respiratory function.^5–8^ The original model of Wells^3^ proposed that droplet dispersion relates to the time a droplet spends in the atmosphere with competing mechanisms of settling, advancement, and evaporation.

The recent literature has analyzed a variety of theoretical and real-life situations to assess the virus transmission potential from a fluid-dynamic standpoint^9,10^ considering the motion of droplets and aerosols.^11–14^ Numerical distance studies of the human respiratory function in Refs. 15 and 16 reported droplet motion up to 2 m axially, with a higher travel distance seen for sneezes^17–19^ and a significant count and distance reduction potential with face coverings.^20–24^ The sensitivity to key ambient parameters, namely, temperature, air humidity, and ventilation flows, was modeled in Refs. 25–27, while the highest infection probability was linked to small droplets in the size range of 10–50 *µ*m.^28^ In addition, chemical studies tested the interaction of droplet drying and precipitation by utilizing an inert nano-colloidal system.^29,30^ The majority of real-life transmission studies focused on classroom,^31^ restaurant,^32^ and public transport^33,34^ settings while investigating the impact of HVAC systems,^35^ opening windows,^36^ and installing glass barriers on the aerosol transport and recommending good sanitization practice, as well as the use of air purifiers.^37,38^ The effect of emitted injection rates in a music classroom was analyzed from singing,^39^ wind instruments, pianos, and improvement recommended with face coverings and portable air purifiers.^40^ Simulations of public restrooms^41^ documented critical concentration levels of aerosolized micro-organisms in the size range of 0.3–3 *µ*m, with limited improvement by using the toilet lid.^42^ Therefore, outdoor environments would provide significant advantages over indoor environments, such as improved ambient ventilation,^43^ little or no spatial confinement,^44,45^ and frequently increased distance between individuals.^2,46^ Validation studies have detailed the dynamics and sensitivity of airborne virus transmission^47–51^ and the dispersion of aerosols under poor ventilation^52^ and provided the best practice to the measurement with handheld particle counters.^53^

Previous investigations from the authors of this paper have further characterized the classroom setting with numerical methods.^54^ A follow-up Computational Fluid Dynamics (CFD) study covered the sensitivity to different classrooms and virus variants,^55^ recommending the use of improved ventilation combined with air purifiers and face coverings. Additionally, a significant impact of human physiology factors was documented by varying saliva properties and geometric features in the nasal and buccal respiratory tracts.^56^ Second, experimental investigations from the authors have confirmed the high sensitivity to the saliva fluid properties^57^ and provided an assessment of safe social distance for human speech and coughing as a function of three types of face coverings.^58^ These investigations form the foundation for the present study, which builds upon these findings and is targeted to expand the data to sneezes, while considering the sensitivity to the key ambient parameter, which is the air humidity.

The present work is focused on the airborne droplet and aerosol emission paths while analyzing the worst-case event of the human respiratory function, a violent sneeze event without any face covering in use. This paper starts with the discussion of the difference between droplets and aerosols and what it means to transmission. This paper then discusses the experimental and numerical studies used in the effort. In Sec. IV, the simulated results are compared and validated with respect to measured velocities and exposure. Finally, the validated CFD model is used to simulate differing conditions of relative air humidity. Quantifying the effect of this ambient parameter could be key to further the understanding and control of airborne transmitted viruses, such as COVID-19.

## II. DROPLETS AND AEROSOLS AND THEIR PHYSICAL CHARACTERISTICS

**FIG. 1.**
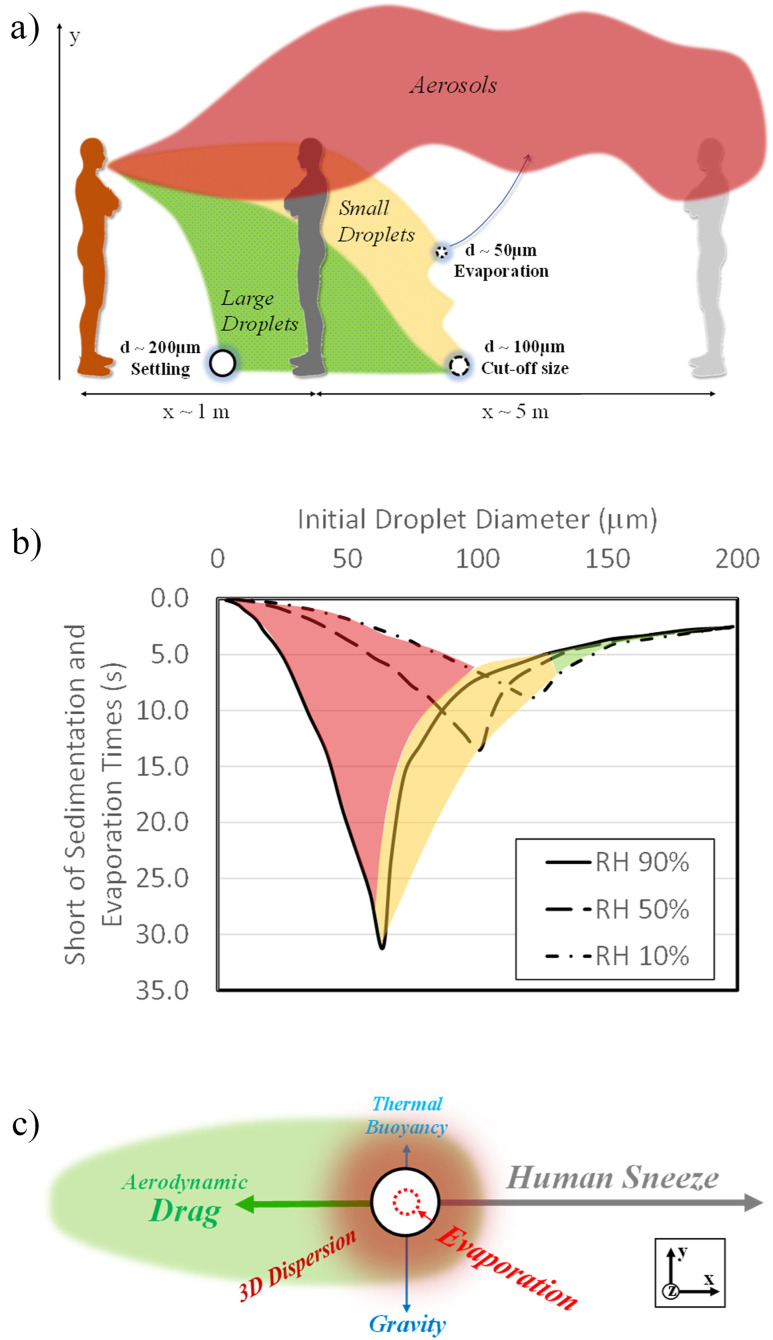
(a) Diagram of airborne droplet/aerosol transmission paths according to Ref. 2, (b) Wells curve^3^ with the influence of air humidity,^1^ and (c) force balance chart outlining the motion of a single droplet in space.

Fundamental historic studies by Wells,^3^ Jennison,^4^ and Duguid^59^ have provided the foundations of today’s understanding and definitions of airborne droplet motion. While relatively simple experimentation methods were used to obtain data, relying mostly on high-speed stroboscopic photography,^3,59^ the outcomes documented maximal expulsion velocities below 50 m/s and travel distances below 1 m,^4^ while the maximal aerosol count was quoted at 30 000 000, with up to 1 000 000 carrying bacteria, and only 52 000 droplets in total.^59^ Key challenges of these historic studies were the limits with optical resolution and quantitative detection capabilities of aerosols at diameters below 1 *µ*m. The modern literature has measured and reported significantly higher numbers for the total expelled number, travel distance, and expulsion velocity of aerosols during a violent sneeze.^60^ The characteristic airborne transmission paths^2^ are provided in Fig. 1(a). Shown are the less critical, ballistic trajectories of the large droplets that settle [green-shaded area in Fig. 1(a)] along with aerosols, which could travel long distances^2^ [red-shaded area in Fig. 1(a)], and small droplets that evaporate [yellow-shaded area in Fig. 1(a)]. The two *d*^2^-relations for the evaporation time (*τ*_*Evap*_) and the settling time (*τ*_*Sed*_) for droplets are defined, respectively, as follows:^2,61^

$$\tau_{\text{Evap}}∼\int_{D_{0}}^{0}-D\left(t\right)dD∼D_{0}^{2},$$ (1)

$$\tau_{\text{Sed}}=\frac{y_{0}}{\frac{{gD}_{0}^{2}\left(\rho_{\text{d}}-\rho \right)}{18\rho \mu }}∼\frac{1}{D_{0}^{2}}.$$ (2)

**FIG. 2.**
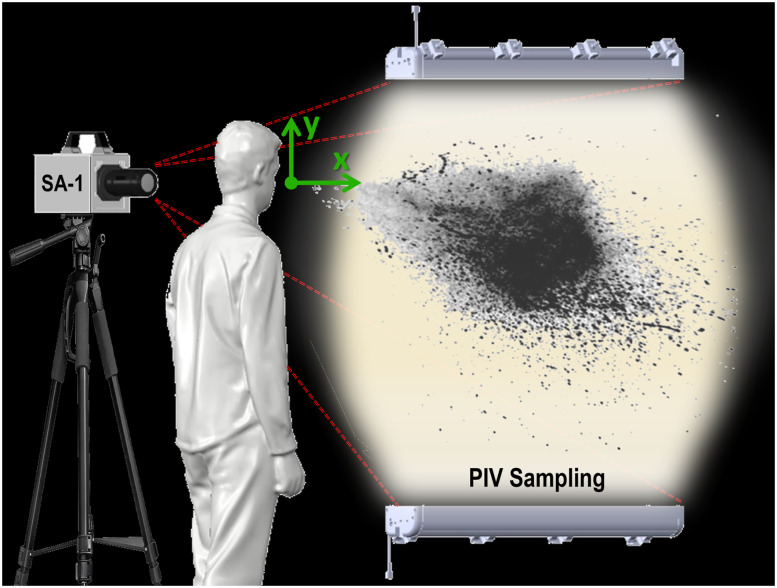
Diagram of the experimental setup with an original imagery sample and labeled equipment.

These relations outline the physical fundamentals for two trendlines of the Wells curve,^3^ shown in Fig. 1(b). These times correspond to the time required to fully evaporate into its underlying precipitates and the time required to settle in quiescent air from one’s mouth (roughly 2 m), respectively. The critical droplet size, the so-called cut-off size, was first postulated by Wells^3^ and documented in detail experimentally in Ref. 1, describing the influence of evaporation and sedimentation. A real-world system and its practical applicability depend on further effects, such as relative air humidity, shown in Fig. 1(b). These plots typically contain the droplet diameter along the x-axis and a (reverse) time-scale along y. Three highlighted droplet size fractions are shown. The red-shaded area (*d* ≤ 50 *µ*m) defines small droplets, a fraction that would certainly evaporate prior to complete sedimentation to the ground from a base level (y = 2 m). In contrast, the green-shaded area (*d* ≥ 100 *µ*m) indicates the large droplet fraction. The latter would certainly fall completely with minor contribution of drying due to their small surface-to-volume ratio. The yellow-shaded area (50 ≤ *d* ≤ 100 *µ*m) is affected similarly by both processes and could complete either falling or evaporation first depending on the ambient conditions. The cut-off size reported in Fig. 2(a) in Ref. 3 is 80 *µ*m. Figure 2(b) also shows analyses of the significant influence of the relative air humidity variation in the cut-off size from a recent study.^1^ In very dry conditions at humidity levels near 10%, droplets with cut-off sizes up to 125 *µ*m are shown to fall due to the low evaporation resistance of fully dry air. In humid conditions (90% relative humidity), the cut-off size was found at 60 *µ*m. Fully saturated, moist air would theoretically not allow droplets of any size to evaporate; hence, a further reduction of that threshold could occur in real systems. Low temperatures and high relative humidity were shown to reduce the cut-off size, allowing more droplets to settle to the ground prior to complete drying.^15^ Generally, higher air humidity levels provide more resistance to evaporation, resulting in longer evaporation scales and a shift of the cut-off size toward smaller diameters [Fig. 1(b)].

Further decomposition of the system physics is provided in Fig. 1(c), showing the diagram of underlying forces onto a single droplet observed. The size of the labels is assimilated to the individual contribution of force magnitudes. The coordinate system is defined in Fig. 4, with x being the positive forward motion and y being the vertical upward axis. The initial motion of the droplets is shown aligned with the positive x direction due to the strong forward expulsion from the mouth. Drag forces come into effect due to aerodynamics and could be amplified by additional drag in case a face covering is used. The aerodynamic interaction is worth additional discussion as it involves the relative velocity of the droplet with respect to a turbulent plume; hence, the interaction involves horizontal (initially), vertical (while settling), and random fluctuations (due to turbulence). Forces along the y and z directions depend strongly on the droplet diameter and the ambient conditions. For large droplets, gravity would influence the resulting movement along a ballistic trajectory curve significantly. As reported in Ref. 1, a cut-off size of about 80 *µ*m occurs in conjunction with the medium–high relative humidity of practical systems. Larger droplets would reach the ground, whereas droplets below the size of 80 *µ*m would fully evaporate prior to complete settling. Additionally, elevated relative air humidity could inhibit the droplet evaporation rates. Three-dimensional diffusive and dispersive effects, as well as any wind or venting-induced flow movement, could redirect the smaller droplets along the ±*z* directions and distract them from their ideal ballistic trajectory, which may lead to measured inconsistencies when sampling is done along the x and y coordinates. Both aerosols and smaller droplets could also be affected by a certain amount of thermal buoyancy, allowing the aerosols to stay suspended in air for minutes before sedimentation.^1^

In summary, the resulting system behavior shows a strong sensitivity to the size of the droplet or aerosol. A smaller droplet diameter would reduce drag forces and sedimentation rates while enhancing three-dimensional diffusive and dispersive effects and other movement dictated by ambient system conditions. Additionally, smaller droplets evaporate faster due to their smaller volume and greater surface-to-volume ratio. A combination of the effects mentioned above explains the complexity of tracking the transmission hazard in systems with high aerosol percentage. Therefore, the present study is defined as a numerical and experimental investigation of the worst-case scenario of human droplet and aerosol emission, a violent sneeze without any face covering in use. The focus is set on the effect of relative humidity since this effect was reported to be among the key influencers on the real-life system. A state-of-the-art commercial CFD tool, Star-CCM+,^62^ is used with a validated droplet model,^63^ providing an Eulerian–Lagrangian, multiphase model of droplets transported from a modeled human sneeze.

## III. METHODS

### A. Experimental aerosol and droplet sampling

The experimental setup used for the study is based on the work of Reyes *et al.*^57,58^ and is depicted in Fig. 2. The setup consisted of a 5MP Photron SA-1 sensor on a tripod and two large static light sources oriented to span an x, y field of view (FOV). The camera captures droplet and aerosol light scatter in a FOV of ∼1.5 × 1.5 m^2^ at a frame rate of 125 fps. The camera was aimed at an opaque, blacked-out curtain to serve as the background, while the test person sneezed perpendicular to the lens axis. All sneezes were recorded under minimal ambient light pollution and in a dust-free environment to minimize ambient particulate noise. The temperature of the room was maintained at 20 °C with 28% ambient relative humidity. The processing was done with ImageJ^64,65^ and MATLAB.^66^ ImageJ served as an initial evaluation method to assess the footage quality, followed by more streamlined processing in MATLAB. Brightness and contrast were digitally enhanced, and the images were binarized (converted to black-and-white) to improve the edge detection of droplets and isolate the clouds of aerosols. The droplet processing provided the droplet diameter, area, count, and eccentricity. To analyze the aerosols, the binarized image was subtracted from the grayscale image to eliminate the droplets and provide an image focused on the aerosol cloud (of subpixel observations). The experiments were used to develop the distance dependent exposure map (DDEP),^57,67^ which indicates exposure to droplets and aerosols at a specific distance over time for various elevations. The exposure map was evaluated at distances of 0.6 and 0.9 m.

The x, y data from the imagery were processed in PIVlab^68,69^ to obtain velocity magnitude. PIVlab is a MATLAB toolbox to process particle imaging velocimetry (PIV) data using the known field of view dimensions and fixed camera frame rate. The flow domain was discretized into smaller interrogation windows, utilizing progressively smaller windows of 128-64-32 pixels to detect the “pairs” of droplets/aerosols between images. The method used to generate the velocity vectors is Fast-Fourier-Transform (FFT) correlation, which is executed automatically in the PIVlab code. The local intensity differences between transient image frames were processed and converted to vectors of velocity magnitude. Vertical line probes for a matrix of nine test points with respect to time and space were extracted. The matrix contained three axial distances 0.3, 0.6, and 0.9 m from the test subject, which were all evaluated for three discrete time steps 0.16, 0.32, and 0.48 s, respectively. The quality of the line probes was enhanced with a filter by averaging over ±2 frames in time and ±2 pixels in space. The observed fluctuation within these frames is quoted as uncertainty and indicated with error bars in Figs. 5 and 6. The physical source of error can be linked to the black curtain cloth, which provided a quality limit due to some wrinkles and reflections. Overall, the experimental results are used to validate the simulation and provide insight into real human sneeze events.

### B. Computational fluid dynamics model

CFD simulation was developed to extract additional insight into the character and drivers of droplet/aerosol transport. The effort is utilizing a commercial CFD code, Star-CCM+, with an Eulerian–Lagrangian multiphase model. The physical scenario is modeled as an Eulerian multicomponent gas phase that couples to liquid Lagrangian saliva droplets (one-way in mass and momentum and two-way in evaporation processes, including energy and H_2_O phase change), as described in previous work.^56,70^ Consequently, the gas phase is solved through Eulerian mass, momentum, energy, and species concentration equations, which are represented by the following partial differential equations:^71–73^

$$\frac{\partial \rho }{\partial t}+\nabla \cdot \left(\rho {\hat{u}}_{i}\right)=0,$$ (3)

$$\frac{\partial \rho {\hat{u}}_{i}}{\partial t}+\nabla \cdot \left(\rho {\hat{u}}_{i}{\hat{u}}_{j}\right)=-\nabla \hat{p}+\rho \vec{g}+\nabla \cdot \bar{\bar{\tau }},$$ (4)

$$\frac{\partial \rho c_{\text{p}}\hat{T}}{\partial t}+\frac{\partial \left(\rho c_{\text{p}}\hat{u}\hat{T}\right)}{\partial x_{\text{j}}}=\nabla \cdot \left(k\nabla \hat{T}\right)+\left(\bar{\bar{\tau }}\cdot \nabla \right){\hat{u}}_{i}+S_{\text{e},evap},$$ (5)

$$\frac{\partial \rho Y_{\text{n}}}{\partial t}+\frac{\partial \left(\rho {\hat{u}}_{i}Y_{\text{n}}\right)}{\partial x_{\text{j}}}=-\nabla \cdot \left(\rho D_{\text{n}}\nabla Y_{\text{n}}\right)+S_{\text{Y},n,evap}.$$ (6)

In the aforementioned equations, *ρ* is the gas phase density, $\hat{u}$ is the velocity field (with subscripts *i* and *j* being the velocity components), $\hat{p}$ is the pressure field, and $\bar{\bar{\tau }}$ is the shear stress tensor of the Detached Eddy Simulation (DES) model.^74,75^ DES aims to use large-eddy simulation (LES) for turbulent scales that the mesh and time step size can support while reverting to the underlying unsteady Reynolds Averaged Navier–Stokes (URANS) model of Spalart–Allmaras simulation^76^ elsewhere. Additionally, high-Reynolds number wall treatment is used and appropriate y^+^ values are used in the prism layers (i.e., y^+^ ∼ 100 for the highest intensity time in the sneeze). The equations couple to an incompressible state equation, $\rho =\frac{P_{\text{o}}}{R\hat{T}}$, where the temperature varies to account for thermal buoyancy. The gaseous phase is a multi-species model captured through the conservation of species mass. For a gas species, *n*, the mass fraction, *Y*_*n*_ in Eq. (6), is tracked for four species, namely, nitrogen (N_2_), oxygen (O_2_), carbon dioxide (CO_2_), and water vapor (H_2_O). The ambient conditions for N_2_, O_2_, and CO_2_ were defined at mass fractions of 0.7, 0.2, and 0.05, respectively, whereas the remaining H_2_O mass fraction varied with time due to the phase change in liquid droplets. The coupling to the liquid phase occurs through the energy and mass fraction source terms from evaporation, given as *S*_*e*,*evap*_ and *S*_*Y*,*evap*_ in Eqs. (5) and (6), respectively. The liquid phase modeling evaporating Lagrangian saliva droplets interfaces with the gaseous phase using a similar approach to our previous modeling^57^ and advances on the work in Ref. 56 in that evaporation is included. The multicomponent droplet evaporation model regards droplets to be internally homogeneous, constituted by the vaporable ideal mixtures of chemicals along with the inert components that remain without vaporization.^77^ The droplet mass change rate of each component due to evaporation ${\dot{m}}_{pi}$ is given by

$${\dot{m}}_{\text{pi}}=-\epsilon_{\text{i}}g^{*}A_{\text{s}}\;\ln\left(1+B\right).$$ (7)

Here, *B* is the Spalding transfer number and g* is the mass transfer conductance. Index *i* represents each component of the mixture of components, and *ϵ*_*i*_ is the fractional mass transfer rate.

The model includes aerodynamic drag,^78^ shear-induced lift, buoyancy, weight, pressure gradient forces, and liquid evaporation through the Ranz–Marshall correlation^79^ that couples to the water-vapor species. The relative humidity is varied by changing the ambient water vapor content. The model accounts for primary and secondary orders of liquid breakup according to the Taylor Analogy Breakup (TAB) model definition in Ref. 63. It is important to note that the TAB model is developed in the context of Newtonian liquids, while saliva is viscoelastic. Our recent results^80^ indicate that viscoelastic droplets are likely to have reduced droplet breakup with respect to the present results. Overall, the model is sufficient to study the impacts of evaporation on droplet dispersion, sedimentation, and suspension. The droplets were assumed to have 10% of the volume as a non-evaporating component, which was defined as a placeholder for substances such as minerals and viral particulate.^5^ To save computational cost, the parcel concept was used to solve the droplet fraction by clustering droplets with the near-identical position and velocity. To calculate the position and velocity of droplets, a first-order integration method is utilized that is coupled to the Eulerian gas phase. These droplets are used to quantify the spatiotemporal quantity of droplets and aerosols. The resulting equation set is solved in the context of a segregated, SIMPLE-C-based algorithm using second-order numerical accuracy (in space and time). The details of the overall gas-phase model and its governing equations are described in previous work.^56^

**FIG. 3.**
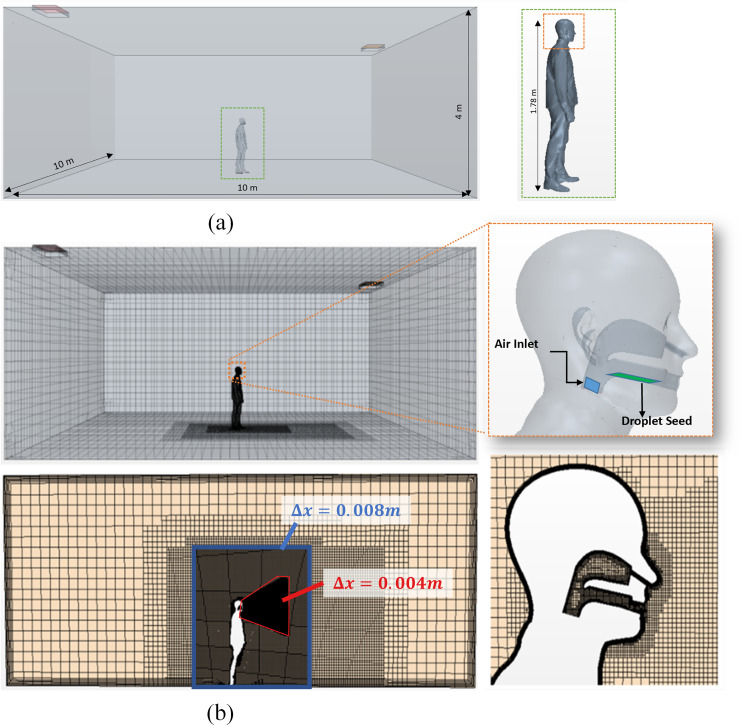
(a) Domain and schematic of gas-phase inlet and liquid droplet injection: geometry of the human sneeze model and (b) local grid refinement at and downstream of the mouth for medium mesh: computational mesh (medium) used for sneeze simulations.

The geometry modeled in the computational domain is indicated in Fig. 3(a) and approximates a human male in the center of the room. The human is positioned at the center of the domain and is 1.78 m tall. The room shape is a cuboid of size 10 × 10 m^2^ and a height of 4 m. The computational mesh is indicated in Fig. 3(b) and uses an octree-based, Cartesian meshing scheme. The mesh also includes prism layer refinement on the no-slip walls on the floor and on the surfaces of the person, including the clothes, bare skin (face/neck), and internal surfaces associated with the upper respiratory tract (URT). Six prism layers were used with a total thickness of 2 mm and a stretch factor of 1.5, resulting in a Δs = 0.15 mm cell layer at the wall. Furthermore, as indicated in Fig. 3(b), the medium mesh was refined in the blue (Δx = 8 mm) and red (Δx = 4 mm) regions where the sneeze was expelled to accurately capture those dynamics with the cell sizes indicated in Fig. 3(b). The coarse and fine mesh grids were obtained by multiplying and dividing these edge lengths by a factor of 2, respectively. It is estimated that the ratio of turbulent-resolving mesh size (Δx) to the Taylor microscale (*λ*) ranges from $\frac{\Delta x}{\lambda }=0.05$ during the high-speed puff event to $\frac{\Delta x}{\lambda }=0.9$ during the convection phase. Overall, the mesh supports a resolution applicable to DES-level turbulent simulations. As shown in Fig. 3(b), the mouth cavity was described for the medium grid with Δx = 2 mm cubical cells in the cavity of the upper respiratory tract and refined to Δx = 1 mm along the nose, the lower respiratory tract, and any domain at a proximity gap size below the wall distance of 5 mm.

The air inlet shown in Fig. 3 acts as a velocity inlet region with spike input with a velocity magnitude of v = 48 m/s for an initial 1 ms and a subsequent drop to 0 m/s exponentially. A part injector placed at the base of the mouth shown in Fig. 3 (droplet seed) injects the Lagrangian saliva droplets. The size distribution of the saliva droplets follows normal distribution with a mean particle diameter of 0.202 *µ*m and a standard deviation of 0.01 *µ*m. The volume flow rate for the Lagrangian fraction peaks at 4 l/s based on the inlet velocity spike profile with a maximum value 50 m/s. Initial temperature and pressure conditions of the modeled surrounding are set to standard conditions (T = 23 °C and *p* = 101 and 325 Pa). The thermal gradients were induced by defining a room temperature and inlet/wall/ground temperature of 23 °C, a body temperature of 29 °C, a buccal surface and breath temperature of 37 °C, and a face temperature of 33 °C to approximate human body temperatures. In order to understand the accuracy of the results, a mesh sensitivity study of the present model was carried out to study this from the perspective of velocity and dispersion. This study expands on the model of our previous work.^56^ For this analysis, three different cases of mesh sizes were used. The “medium” mesh is indicated in Fig. 3(b) along with the various cell sizes in the various refinement zones. A “coarse” mesh was developed by increasing the edge length in each zone by a factor of 2.0 in all directions with exception of the prism layer cells. Similarly, the “fine” was generated by decreasing the edge length by a factor of 2.0 relative to the “medium.” The results for the sequence of meshes are evaluated in terms of the velocity and droplet concentration at distances of 0.6 and 0.9 m from the mouth. The results are presented in Sec. IV and indicate that the medium mesh is asymptotic in both velocity and concentration.

## IV. RESULTS AND DISCUSSION

Section IV A investigates several metrics that were extracted to benchmark the simulation against our experimental data. In Sec. IV B, the validated model was applied to study the impact of varying relative air humidity.

### A. Benchmarks of CFD using experimental data

#### 1. Exposure maps

**FIG. 4.**
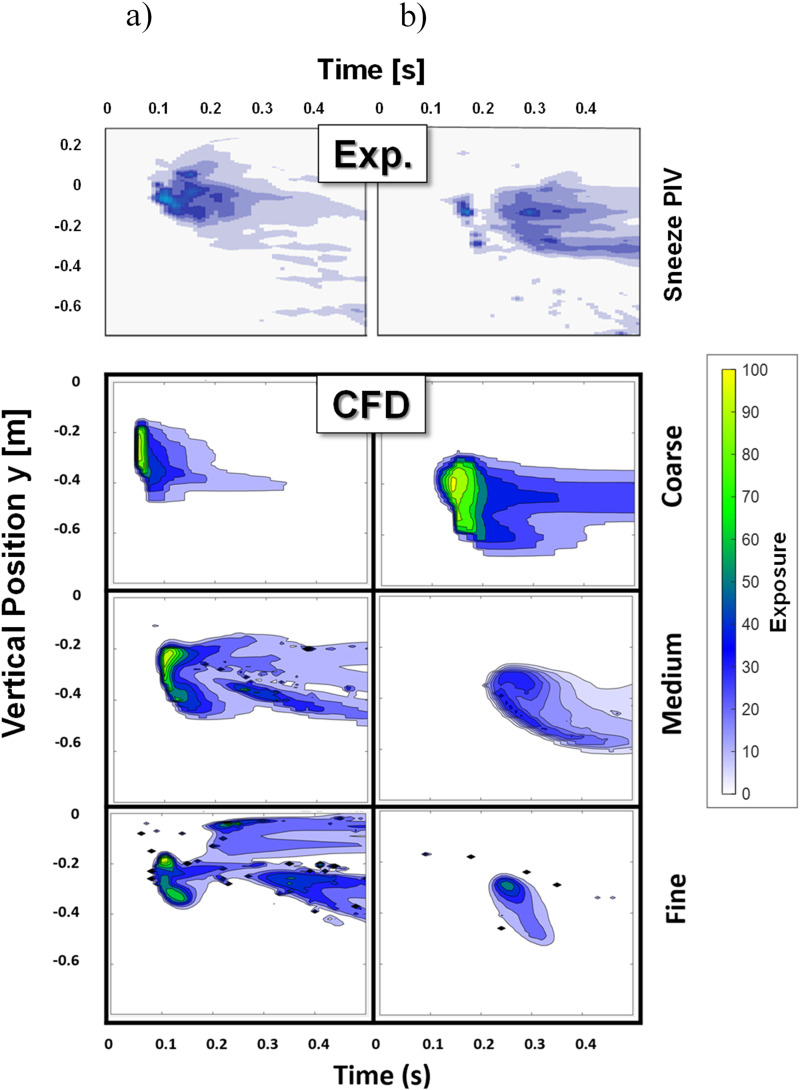
Exposure maps at axial distances: (a) x = 0.6 m and (b) x = 0.9 m. Top: exposure map reconstruction from experimental imagery. Bottom: CFD results at three grid refinement levels.

Figure 4 shows the extraction of “exposure,” which is a measure of high local sneeze emission occurrence. The concept of such a map was developed in previous work^56,57^ and indicates overall exposure to aerosols and droplets at a given distance and over time. At a specific elevation, the overall value is used to qualitatively estimate the CFD model accuracy of the dispersion of droplets and aerosols. In Fig. 4, the exposure maps at 0.6 m (left column) and 0.9 m (right column) are plotted for the experiment (top) and the CFD results at various mesh resolutions (lower). In each of the exposure maps, the x-axis is the time in seconds and the y-axis is the vertical position reference centered at the mouth of the human body. For the experiment (first row), data were extracted from experimental imagery, with background subtraction and the minimum level shown being 2.5% of the peak exposure. Similarly, the simulations (rows 2–4) were processed with background subtraction. Both simulations and experimentation data show the maximal local exposure after 0.6 m, found in the mouth near-field and slightly below the horizontal head level. At 0.9 m, the trends are similar but appear to be at reduced exposure levels due to ambient factors. Overall, the correlation between CFD and experiments in Fig. 4 indicates reasonable agreement considering the high degree of expected variation from sneezes, persons, etc. While such a comparison is qualitative in nature, it provides indication as to how well the CFD can predict dispersion during a sneeze. Despite the minor deviations, the CFD was able to pick up a similar trend of reducing exposure with increasing time and with increasing axial distance.

#### 2. Human sneeze timescales

**FIG. 5.**
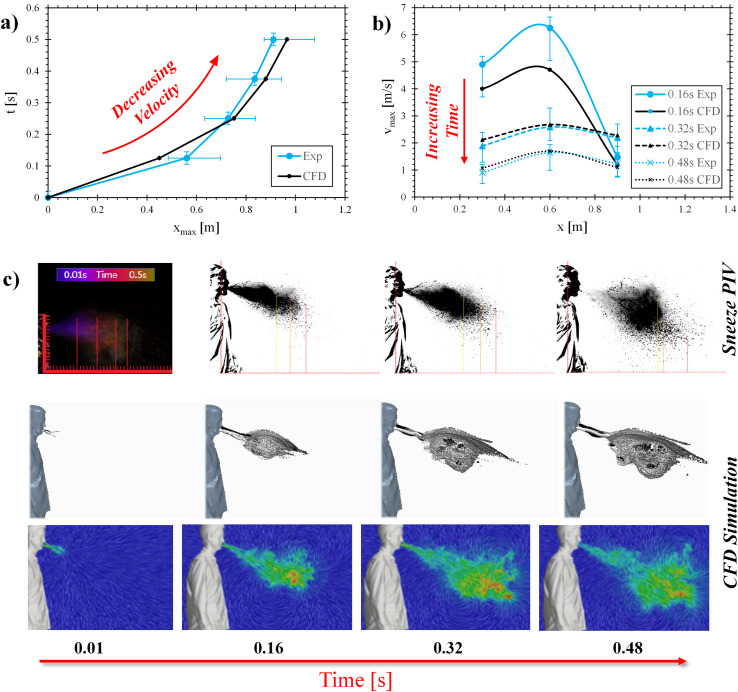
Characteristic human sneeze timescales: (a) time vs maximum axial protrusion distance, (b) maximum velocity, sampled for three time steps along three axial distances, and (c) validation data: experimental imagery vs modeled droplet and velocity field contours.

Global values were obtained via an experimental case where airborne droplets from the subject are tracked against a contrast-enhanced, isolated background (described in Sec. III B) and a CFD centerline droplet and velocity field contour plot. The experimental distribution is indicated with values extracted at the furthest axial distance points of 95% [red line in Fig. 5(c)], 50% (orange line), and 5% (yellow line) measured droplet exposure.

Figure 5(a) shows the global relationship between the maximum axial travel distance and time over a period of half a second after droplets are released from the subject. Figure 5(b) highlights velocity profiles along the axial sample distance at three distinct time points of 0.16, 0.32, and 0.48 s after release. Both figures can be visually supported by the collection of the respective time-stills provided in Fig. 5(c), showing the droplet cloud in the CFD to remain in good correlation over time with the experimental test data. Maximum deviations between simulated and experimental data amount to 9% in Fig. 5(a) and 17% in Fig. 5(b). The variance of the PIV data ranges between 4% and 15% due to the variability between persons and sneezes and uncertainty due to background quality limitations. As can be seen from Figs. 5(a) and 5(b), experimental findings show droplets to be ejected slightly farther during the first 0.16 s after release, compared to computed results. After that time, the experimental data show a slightly greater dissipation along the axial velocity coordinate approaching 0.5 s, compared to the CFD. While the high velocity of droplets in the initial stages of the data causes a deviation between the experimental and CFD cases [Fig. 5(b), t = 0.16 s], the two tests trend toward the same velocity levels in the far-field (t = 0.32 s and t = 0.48 s). Overall, this near-field CFD validation shows a good overlay. The turbulent vortex roll-up structure was accurately captured in the model, which substantiates an accelerated velocity decrease between t = 0.32 s and t = 0.48 s.

#### 3. Vertical velocity profiles

**FIG. 6.**
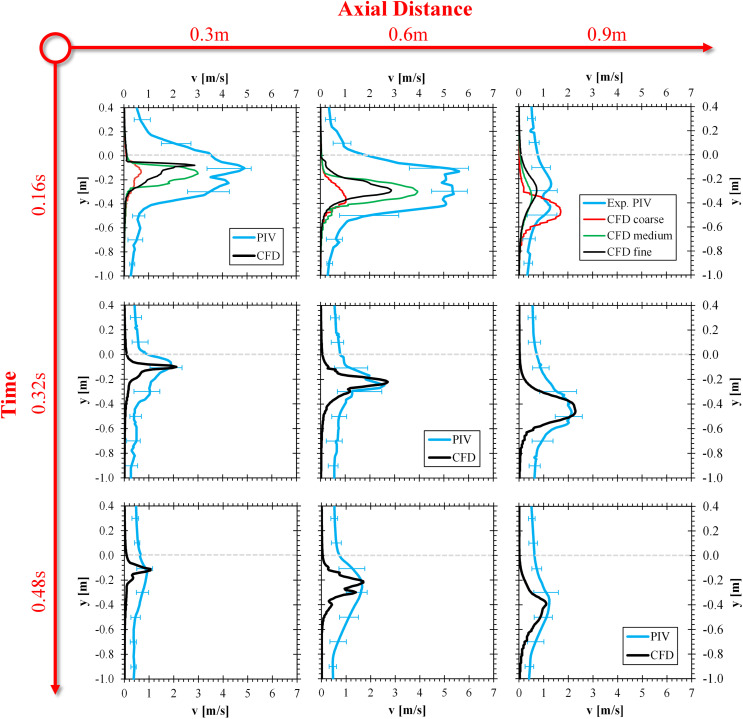
Vertical velocity profiles for a human sneeze extracted after 0.3, 0.6, and 0.9 m along the axial coordinate for three points in time 0.16, 0.32, and 0.48 s. Experimental data were recorded with PIV, and modeled data were processed with line probe extraction.

Section IV B describes a 1D overlay of simulated vertical velocity profiles against experimental PIV data. CFD data for the velocity magnitude were extracted along resolved, vertical line probes, located 0.3, 0.6, and 0.9 m downstream of the subject’s mouth. Time steps of 0.16, 0.32, and 0.48 s analyzed the transient droplet emission. Similar data were extracted from the experimental PIV, filtering along a five-pixel wide line probe window, while considering the three respective axial positions and time steps (cf. Sec. IV A). In addition to the high initial sneeze velocities, which could be greater than 150 m/s according to numerical estimates, the PIV results in Fig. 6 show maximal velocities of 5 m/s (x = 0.3 m) and 6 m/s (x = 0.6 m), both at 0.16 s, which is in agreement with the simulated data. Some of the local velocity hotspots shown in Fig. 5 were picked up with the PIV data in Fig. 6, located at about 80% of the maximal, axial sneeze distance. In the temporal near-field (t = 0.16 s), the experimental PIV data show a considerably broader distribution along ±y, compared to the simulated outcomes. Reasons for this could include the enhanced three-dimensional effects (cf. Fig. 4), inducing stronger near-field dynamics and some transient mouth movement during a sneeze, which was not accounted for in the CFD model. A certain noise level was picked up with PIV for the experimental sampling points, with velocity magnitudes of 0.3–0.4 m/s, compared to quiescent surrounding air in the CFD model. In summary, Fig. 6 shows that a good overlay between simulated and experimental vertical profiles was achieved, with deviations at t = 0.16 s documented and a very good overlaying trend shown for the later time steps t = 0.32 and 0.48 s. Additionally, the grid sensitivity study was repeated in Fig. 6 for all distances at 0.16 s, suggesting the medium grid refinement level to be of sufficient accuracy to capture the human sneeze velocity profile.

### B. Parameter study: Influence of relative air humidity

The subsequent sections (IV B 1 and IV B 2) analyze the influence of relative air humidity (RH) based on the validated CFD model. Section IV B 1 compares the model outcomes with results from the study by Vuorinen *et al.*,^1^ and Sec. IV B 2 provides an assessment of the associated airborne transmission risk.

#### 1. Correlation of modeled data with the Wells curve

**FIG. 7.**
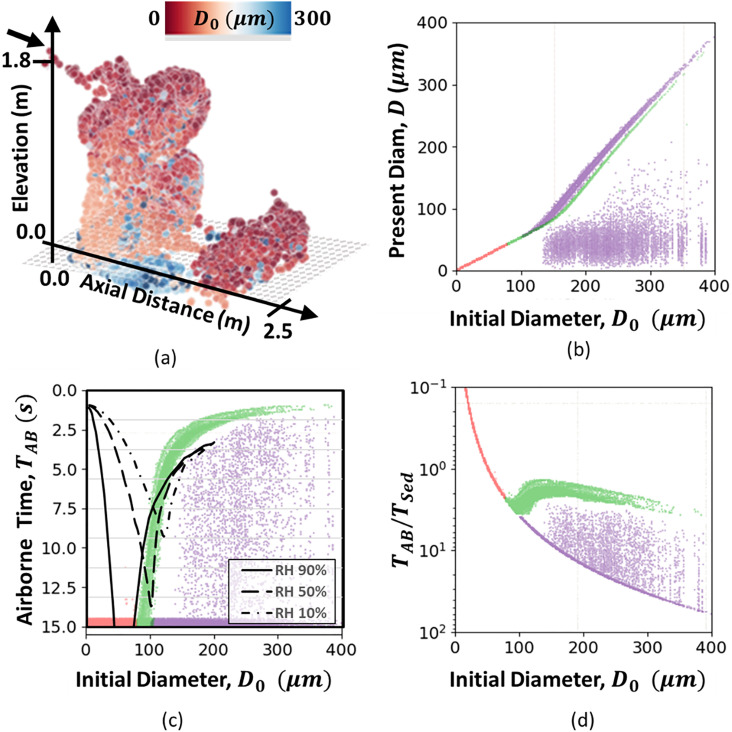
Evaluation of Wells curve details after t = 15 s for a RH of 28%. Green droplets are sedimented, red droplets are suspended, and purple droplets are suspended and were estimated to be sedimented. (a) 3D droplet distribution: 3D plume structure vs initial droplet size, (b) evaporating droplets: sedimentation time vs Wells-curve predictions from Ref. 1, (c) observed Wells curve: current vs initial droplet diameter, and (d) time ratio: suspended and sedimented particle diameters vs ratio of airborne time to expected suspended time.

Based on the validated CFD model, a reconstruction of the Wells curve is investigated in this section. The results from the base case from the experiment benchmark with a RH of 28% are depicted in Fig. 7 and are analyzed using Gluviz^81,82^ to provide insight into the general characteristics of droplets for many parameters. Figure 7(a) shows a three-dimensional distribution of the droplets/aerosols after 15 s from the initiation of the sneeze event. Each droplet is colored by the initial diameter. Note the initially large droplets that appear to remain suspended depicted through blue droplets at high elevations. Explaining this phenomenon demands discussion of evaporation, which is described in Fig. 7(b) and plots the present diameter against the initial diameter at 15 s. Each point is plotted and colored red if it is suspended, green if it is settling faster than evaporation and in-line with Stokes terminal velocity (super-suspension), and purple if it is still suspended but should have settled. The droplets plotted in purple typically indicate significant evaporation—these are the large droplets that typically remain suspended. At this time level, a reproduced Wells curve is provided in Fig. 7(c) and plotted in comparison to the results from the study by Vuorinen *et al.*^1^ Note the excellent agreement with the model character. This figure plots each droplet’s airborne time, *T*_*AB*_ (i.e., recorded time of the droplet in the domain, yet not adhering to the floor), vs the initial droplet diameter. The plot clearly signifies a sedimentation curve; however, the indefinite time associated with evaporated droplets leads to run time. Hence, it is proposed to evaluate the Wells curve as performed in Fig. 7(d), which plots the ratio of airborne time to the expected sedimentation time, $\frac{T_{AB}}{T_{\mathrm{Sed}}}$. This parameter is calculated using the time required to settle using Stokes terminal velocity from y = 1.8 m, given as *T*_*Sed*_ [Eq. (2)]. For this curve, the long sedimentation times of the smaller aerosols in the model (compared to the relatively short CFD run time) yield a curve that replicates the evaporation branch of the Wells curve. Like in Figs. 7(a)–7(c), the red droplets are those that remain suspended, while the green droplets have sedimented to the ground and correlate well with the droplets that have a minimal evaporation [Fig. 7(b)]. Note that in the context of a sneeze, the settling time tends to be up to 3.5 times longer than the time expected for Stokes terminal velocity. The purple droplets indicate a complete underprediction of the settling time, yielding a range of droplets that are suspended in the sneeze for long enough to evaporate. Observing these data, it is clear that the structure and velocity of the violent sneeze lead to a scenario of advancing suspension of many droplets. Of key importance is the viral load of the purple droplet fraction. Under the assumption of constant viral load in the mucus/saliva films, these droplets are more likely to contain more viral particles. Hence, the quanta per particle are higher and can lead to an increased probability for transmission events. In general, the more advanced CFD model indicates that the Wells curve may not be conservative and completely misses factors associated with evaporating large droplets with potentially higher levels of viral particles.

**FIG. 8.**
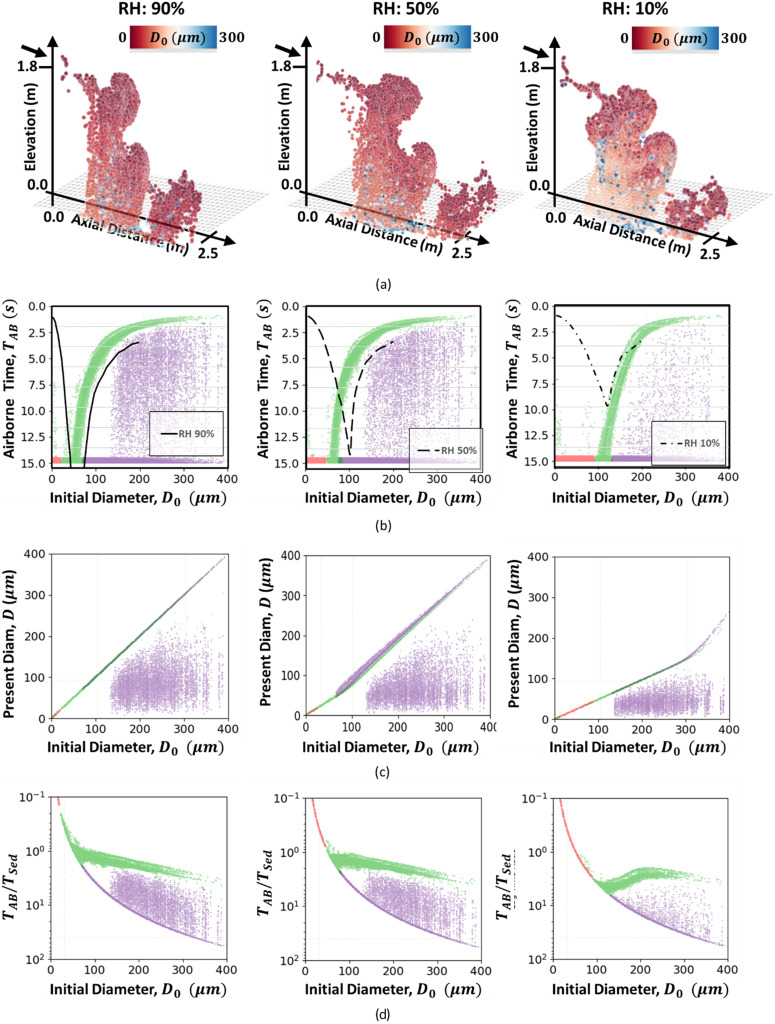
Effect of relative humidity on the sneeze distribution after 15 s: (a) 3D droplet distribution: 3D plume structure vs initial droplet size, (b) observed Wells curve: sedimentation time vs Wells-curve predictions from Ref. 1, (c) evaporating droplets: current vs initial droplet diameter, and (d) time suspended relative to sedimentation time: suspended and sedimented particle diameters vs ratio of airborne time to expected suspended time. Green droplets are sedimented, red droplets are suspended, and purple droplets are suspended and were estimated to be sedimented.

The overall study of the droplets with respect to the Wells curve is further investigated with respect to a varied relative humidity in Fig. 8. Figure 8(a) shows comparisons of the droplets after 15 s. Note that the low relative humidity case (RH: 10%) has more droplets suspended that initiated with a larger diameter. Figure 8(a) visualizes the 3D droplet distribution in the sneeze plume, colored by the initial droplet size. Figure 8(b) compares the suspension time to the Wells curve.^1^ In general, the sedimentation arm (right) is well captured. Note that the model still predicts droplets that are suspended longer than initially assumed, which occurs for all RH values. An indication of the evaporation character is highlighted in Fig. 8(c), which, as expected, indicates that the higher RH values lead to reduced evaporation, as indicated by the higher values of the droplet diameter (after 15 s) with respect to the initial droplet diameter. Finally, the time suspended with respect to the suspension time provided in Fig. 8(d) continues to indicate the character associated with suspended particles. However, it becomes apparent that the dip in the transition (RH: 10% near 100 *μ*m) appears worse for lower relative humidity [see Fig. 7(d)] and behaves more near-unity for higher RH values. This likely indicates that there are sizes that react to turbulent suspension, leading to such an event in bulk.

#### 2. Impact of relative humidity on airborne transmission risk

**FIG. 9.**
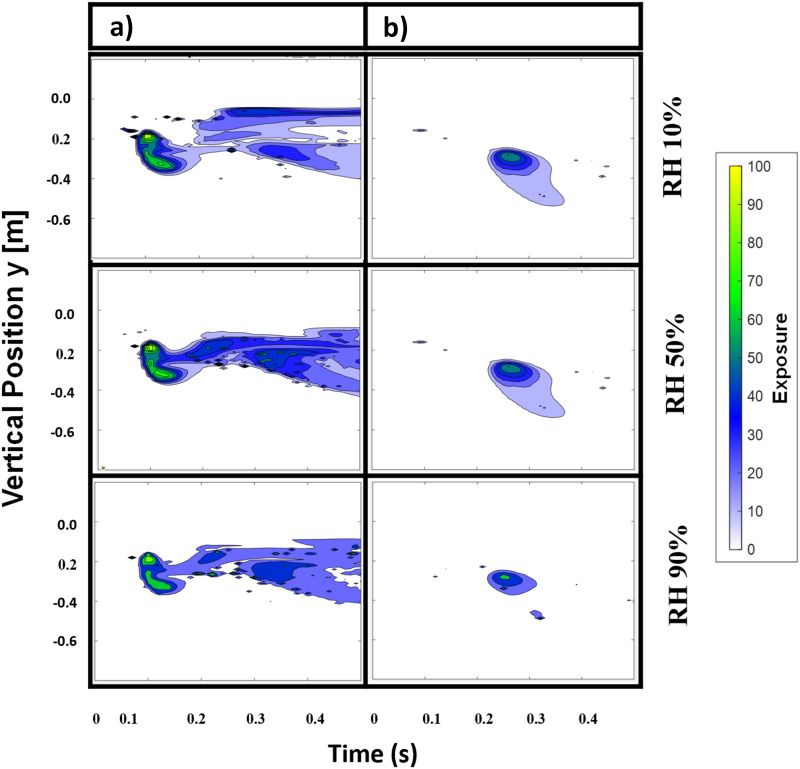
Effect of relative air humidity on the droplet exposure level in the subject’s near-field. The first column (a) and the second column (b) represent the exposure levels at distances of 0.6 and 0.9 m away from the human body, respectively.

Figure 9 shows the exposure maps for different relative humidity levels of the sneezing phenomenon. The x-axis shows the time in seconds, and the y-axis is the vertical position reference, centered at the mouth of the human body. In this figure, exposure maps are aligned in the ascending order for the cases of RH 10%, RH 50%, and RH 90% from top to bottom. Axial distances of 0.6 m (left column) and 0.9 m (right column) were evaluated. At all relative humidity levels, the exposure reduces with the increasing distance from the mouth. Second, a substantial reduction in the exposure level can be correlated with increasing RH. This effect is pronounced at an axial distance of 0.9 m, as only traces of exposure were predicted when the RH is increased from 10% to 90%. The findings provide indication that virus transmission or exposure levels could be reduced by increasing the RH. The documented effect can be linked to the lower rate of droplet evaporation due to the increased resistance at high RH. While a large droplet would not fully evaporate at any air humidity level, the effect described above will likely affect the total airborne time of the small droplet fraction by elongating its evaporation timescales.

**FIG. 10.**
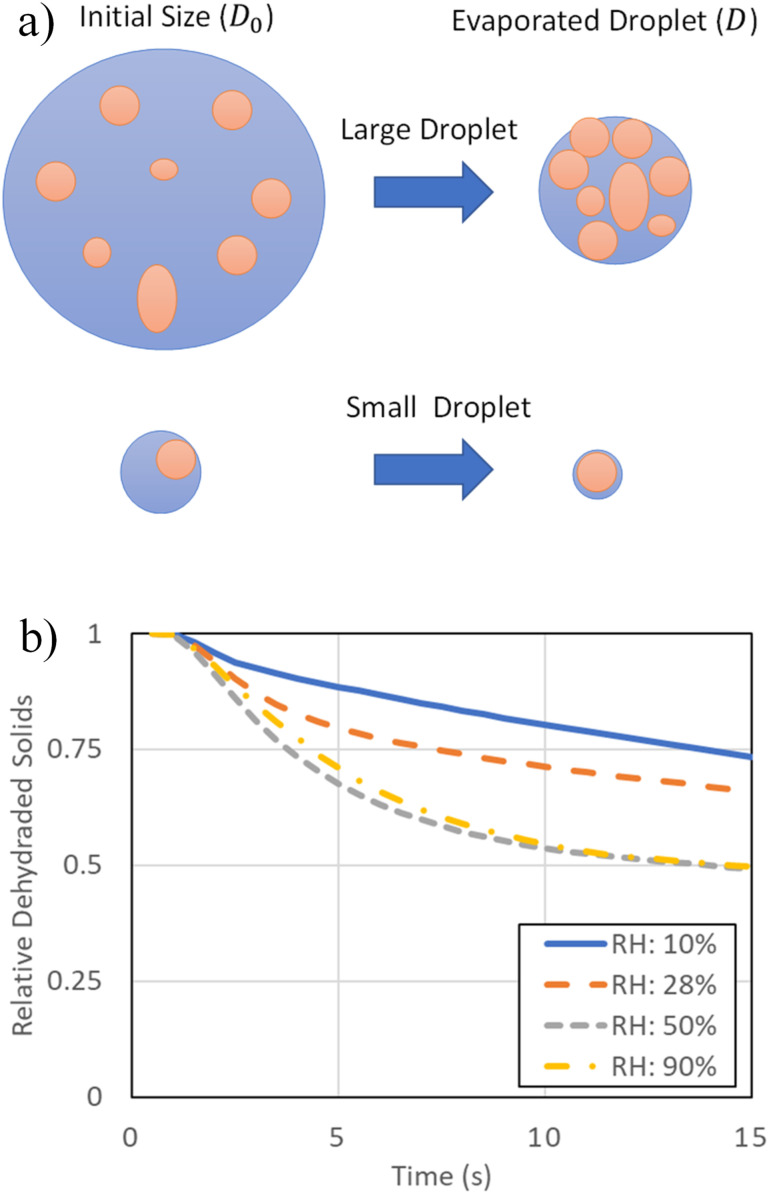
Relative contents of the non-evaporative material from droplets that have remained suspended over time. Such a metric is likely to directly relate to transmission risk. (a) Dehydrated mass from large (upper) and small (lower) droplets and (b) suspended dehydrated solids as a function of RH.

In order to assess the impact of evaporation on transmission risk, the suspended contents of the material that is non-evaporative are evaluated. For the model, it was assumed^5^ that 10% of the volume would not be able to evaporate. The process was developed in the work of Liu *et al.*^83^ and is conceptually indicated in Fig. 10(a). These non-evaporating materials would include minerals and the viral particles associated with transmission. These suspended contents (defined by droplets with heights greater than 0.1 m) are evaluated in Fig. 10(b). These evaluations are studied in the context of a variable relative humidity. In general, it is clear that the low relative humidity (10%) leads to elevated levels of suspended viral particulate. This is a direct result of the larger droplets evaporating to small sizes that can remain suspended. As the relative humidity increases to 28% and 50%, the contents of suspended viral particles appear to decrease by 10% and 33%, respectively. For a relative humidity above 50% (up to 90%), the dehydrated solids remained at a relative percentage of 50%. Such a trend is likely to be more important in the context of larger droplets in the distribution since they would require more time to evaporate. Overall, the results indicate that there is potential to reduce risk by roughly 30% by maintaining relative humidity above 50% and is a critical factor associated with desert and winter climates, where relative humidity can be low.

## V. CONCLUSIONS

This paper has numerically and experimentally investigated the key fluid-dynamic characteristics, which are known to be in high correlation with airborne disease transmission of infectious viral particulate, such as COVID-19. A violent sneeze without a face covering or other physical protection in use was studied to detail the worst-case of respiratory emission potential. The high counts of emitted droplets and aerosols make a sneeze the worst-case of the human respiratory function. The numerical model uses detached eddy simulation and the Lagrangian–Eulerian multiphase model coupling with the Taylor analogy breakup method. The CFD model was found to be relatively insensitive to further grid refinement while providing reasonable correlation with our corresponding experimental data. The data were taken using an optical imagery method and processed to validate spatiotemporal exposure maps, vertical velocity profiles, and emission propagation profiles. The model was further validated against recent literature data from the study by Vuorinen *et al.*^1^ and visualized according to the original model reported by Wells *et al.*,^3^ providing a benchmark dataset to the evaporation and sedimentation bias as a function of time and droplet diameter. The validated CFD model was then applied to visualize parameters that are challenging to measure directly in the experiment. Multi-dimensional droplet and aerosol size distribution maps were provided to quantify the emission propagation profile and outline the amount of relative evaporation and sedimentation bias. Then, the effect of differing ambient surroundings was studied with the variation in the relative air humidity (RH) in the model. The experimental air humidity of 28% was validated, and the model was used to predict characteristics at 10% (desert), 50% (moderate), and 90% (tropical) relative humidity levels. The results show a high sensitivity of the Wells curve to the air humidity. The maximal airborne time was 10 s at 10% RH, 14 s at 50% RH, and just above 20 s at 90% RH. Meanwhile, the cut-off size reduced from 120 *µ*m (10% RH) to 100 *µ*m (50% RH) and reduced further to 70 *µ*m (90% RH). Finally, the amount of dehydrated solids was shown to reduce significantly with increasing humidity and reach a minimum level at and above 50% RH with no further reduction potential. Therefore, the findings support a 33% reduction of the airborne transmission risk in wet climates, or vice versa, the risk of contracting airborne infection is increased by almost 50% in desert and winter climates or in dry indoor environments.

## Data Availability

The data that support the findings of this study are available from the corresponding author upon reasonable request.
